# After abduction: exploring access to reintegration programs and mental health status among young female abductees in Northern Uganda

**DOI:** 10.1186/1752-1505-8-5

**Published:** 2014-05-07

**Authors:** Katherine A Muldoon, Godfrey Muzaaya, Theresa S Betancourt, Mirriam Ajok, Monica Akello, Zaira Petruf, Paul Nguyen, Erin K Baines, Kate Shannon

**Affiliations:** 1 British Columbia Centre for Excellence in HIV/AIDS, St. Paul’s Hospital, 608-1081 Burrard Street, Vancouver, BC V6Z 1Y6, Canada; 2 School of Population and Public Health, University of British Columbia, 2206 East Mall, Vancouver, BC V6T 1Z3, Canada; 3 Liu Institute for Global Issues, University of British Columbia, 6576 NW Marine Dr, Vancouver, Canada; 4 The AIDS Support Organization, Gulu, Uganda Mulago Hospital Complex, PO Box 10443, Kampala, Uganda; 5 Department of Global Health and Population, Harvard School of Public Health, 677 Huntington, Ave, Boston, MA 02115, USA; 6 Simon Fraser University, Burnaby, British Columbia, 8888 University Drive, Burnaby, BC V5A 1S6, Canada; 7 Department of Medicine, University of British Columbia, St. Paul’s Hospital, 608-1081 Burrard Street, Vancouver, BC V6Z 1Y6, Canada

**Keywords:** Reintegration, Abduction, Women, Northern Uganda, Mental health

## Abstract

**Background:**

Reintegration programs are commonly offered to former combatants and abductees to acquire civilian status and support services to reintegrate into post-conflict society. Among a group of young female abductees in northern Uganda, this study examined access to post-abduction reintegration programming and tested for between group differences in mental health status among young women who had accessed reintegration programming compared to those who self-reintegrated.

**Methods:**

This cross-sectional study analysed interviews from 129 young women who had previously been abducted by the Lords Resistance Army (LRA). Data was collected between June 2011-January 2012. Interviews collected information on abduction-related experiences including age and year of abduction, manner of departure, and reintegration status. Participants were coded as ‘reintegrated’ if they reported ≥1 of the following reintegration programs: traditional cleansing ceremony, received an amnesty certificate, reinsertion package, or had gone to a reception centre. A t-test was used to measure mean differences in depression and anxiety measured by the Acholi Psychosocial Assessment Instrument (APAI) to determine if abductees who participated in a reintegration program had different mental status from those who self-reintegrated.

**Results:**

From 129 young abductees, 56 (43.4%) had participated in a reintegration program. Participants had been abducted between 1988–2010 for an average length of one year, the median age of abduction was 13 years (IQR:11–14) with escaping (76.6%), being released (15.6%), and rescued (7.0%) being the most common manner of departure from the LRA. Traditional cleansing ceremonies (67.8%) were the most commonly accessed support followed by receiving amnesty (37.5%), going to a reception centre (28.6%) or receiving a reinsertion package (12.5%). Between group comparisons indicated that the mental health status of abductees who accessed ≥1 reintegration program were not significantly different from those who self-reintegrated (p > 0.05).

**Conclusions:**

Over 40% of female abductees in this sample had accessed a reintegration program, however significant differences in mental health were not observed between those who accessed a reintegration program and those who self-reintegrated. The successful reintegration of combatants and abductees into their recipient community is a complex process and these results support the need for gender-specific services and ongoing evaluation of reintegration programming.

## Introduction

Disarmament, Demobilization and Reintegration (DDR) is an applied strategy generally employed by all UN Peacekeeping Operations and is designed to provide amnesties and benefit or reinsertion packages to those that have been involved in mass atrocities as leverage to sign a peace agreement and disarm [1]. Disarmament (i.e. physical removal of weapons and munitions) and demobilization (i.e. disbanding of armed groups) are well defined and occur relatively immediately in peace processes [1]. Disarmament and demobilization are commonly implemented by national governments and multilateral agencies with the goal to foster conditions for sustainable investment, good governance and ultimately provide stability for transitional justice mechanisms to operate [2].

Reintegration is the process where former combatants and abductees acquire civilian status and support services to reintegrate into post-conflict society [3]. It is a complex and open-ended process between the combatants, abductees and the recipient community that cannot be imposed or centralized. As quoted by De Vries (2011) ‘one cannot ‘programme’ people into accepting one another after years of violence conflict’ [4]. The objectives of reintegration programs are generally to provide support and opportunities to live ‘normal’ lives, become a functional member of society, resume education, gain skills training, and reduce trauma, including anxiety and depression [1]. Participation in armed groups is associated with exposure to extreme violence, and research has shown a higher frequency of trauma exposure and mental illness among abductees compared to their non-abducted peers living through the same conflict [5], however, the specific kinds of mental illness experienced remain an important area for further exploration. Reintegration of combatants and abductees is an essential component for establishing long-term stability for fragile states affected by conflict.

Abduction or forced conscription of children has been documented in armed conflicts in Angola, Burundi, Democratic Republic of Congo, Mozambique, Rwanda, Somalia, Sudan, Sierra Leone and Uganda [6]. In many contemporary African wars, girls and women participate in armed forces, however the majority of ex-combatants are male, and this has led to programs designed predominantly by men for men. Fewer women and girls go through the official UN processes of DDR and continue to face substantial challenges in their physical and psycho-social recovery, including social ostracization, trauma, depression and anxiety [7,8]. Although gender differences are increasingly acknowledged in conflict-affected settings, in many studies the experiences of women and girls are often combined with those of men and boys subsequently limiting inference to female populations. Against this backdrop, young women in general and particularly young abductees continue to face large gender disparities in economic opportunities, access to education, including high rates of female illiteracy [9], all conditions that inhibit successful reintegration. The latest edition of the UN DDR guidelines have specifically called attention to the need for research to further understand and support the needs of women and girls affected by war [10]. The inclusion of gender analyses is an important component of designing successful DDR programs, particularly to support the reintegration phase that takes the most time and is set against the backdrop of the gender inequities that often exist in the pre and post-conflict society [7,11].

### Northern Uganda

DDR programs have featured prominently in the conflict-affected landscape of northern Uganda. The conflict between the Lord’s Resistance Army (LRA) and the Governments’ Ugandan Peoples Defense Forces (UPDF) lasted over two decades where it is estimated that between 25,000 and 60,000 youth between the ages of 13 and 30 were abducted into the Lords Resistance Army (LRA) over the course of the war [12]. Different documents report increased frequency of abduction into the LRA corresponding to historical events during the war including the two government-led military offensives, Operation North (1991) and Operation Iron Fist (2002), and a high profile event in the town of Atiak in 1995, where the LRA massacred over 300 civilian men and boys [13] and abducted hundreds of girls from schools in the surrounding area [14]. The frequency of children abducted over the course of the war was documented by Amnesty International and Human Rights Watch [15,16], however few other studies have investigated the frequency of abduction among their samples and how they correspond to the historical progress of the war in Uganda.

The DDR operation in northern Uganda had the first task of disarming and demobilizing the LRA [4,17,18]. Under the Amnesty Act, any Ugandan citizen involved in the insurgency against the UPDF since 1986 is eligible for amnesty and to participate in DDR programs. The Amnesty Act was reified in 2000, however the Amnesty Commission only began providing certificates and aid to combatants that returned after 2000. When reporters apply for amnesty they commonly register at Reception Centres, a halfway house between rebel life and civilian life [19]. It was reported that NGO and government Reception Centres were able to offer some level of counselling, education and skills training [19]. However, it is estimated that over 25% of returnees did not report to the authorities [19] and only 13% of registered abductees received a follow-up visit from the reception centre [20]. Evidence to support access to reintegration programming, and evaluations of the association between accessing reintegration programs and health outcomes, including mental health, remain an important gap in the literature [11].

To address these converging concerns, the aim of this analysis is to: 1) describe a sample of female former abductees and explore abduction-related experiences including the frequency and duration of abduction over the timeline of the war; 2) determine the proportion of female former abductees that were able to access a reintegration program compare to those who self-reintegrated; and 3) assess differences in mental health status among female former abductees who participated in a reintegration process compared to those who self-reintegrated.

## Methods

### Sample and recruitment strategy

Data for this analysis comes from a community-based cross-sectional study of HIV prevention, treatment and care among 400 young sex workers in northern Uganda (May 2011-January 2012). Study eligibility criteria included: ≥ 14 years and having exchanged sex for money or resources (e.g. food, cell phone air time, jewellery, shelter, childcare etc.) in the previous 30 days. This project was conducted in partnership with TASO Gulu and other community-based youth, women, sex work and health service organizations. TASO is the largest indigenous HIV service organization and at the time of this research, has no formal programming or funding for programming with sex workers. However the program has since expanded efforts in response to noted gaps to reach sex workers and other key affected populations (e.g. men who have sex with men). Extensive outreach and engagement with community, sex workers, and external stakeholders (NGOs, health services, police) were undertaken by a research team. Participants were recruited through peer/sex worker-led outreach (former/current sex workers) to bars and hotels, where sex workers work, as well as community-led outreach to former IDP camps (i.e. Pabbo, Bobi, Awach, Labongogali) together with The AIDS Support Organization (TASO) Gulu clinic. Study questionnaires collected information on demographics, sex work histories and current working environments, intimate partnerships, trauma and violence (including war related violence and abduction), sexual and reproductive health, and HIV prevention, treatment and care. Structured interview-administered questionnaires and voluntary HIV counselling and testing (HCT) were conducted by Acholi research assistants and offered in the Luo language. Consultation sessions with sex workers and service providers were conducted for input on questionnaire development, and piloting with participants. The study was explained verbally in Luo and each participant received a copy of the consent form. Participants provided written informed consent or provided a thumbprint in the case of limited literacy. The analytical sample was restricted to 129 women who self-reported being former abductees of the LRA.

### Variables of interest

#### Abduction related variables

Participants were asked the age and year of abduction, the length of time they remained in LRA captivity, and how they eventually departed. In the absence of a standardized measure, a literature review was conducted to identify common reintegration programs for war-affected populations. This list was also supplemented by discussions with the research study team of Acholi staff. Participants were asked if they had participated in a reintegration process or program including any of the following: traditional cleansing ceremony, receiving an amnesty certificate, registration and lodging at a reception centre, or receiving a reinsertion package. Those who reported that they had not participated in any form of reintegration were classified as abductees who self-reintegrated, the rest were classified as abductees who accessed reintegration programming. A dichotomous variable was derived to compare those who had a child with a LRA member to those who had not. All variables are self-report.

#### Mental health

The mental health status of study participants was assessed using a locally-developed and validated scale called the Acholi Psychosocial Assessment Instrument (APAI) [21]. The APAI was developed using free listing interviews with youth, local adults and key informants to identify and describe signs of mental health syndromes affecting Acholi youth. The APAI scale includes 60 locally identified signs and symptoms on a likert scale from ‘0’ (never) to ‘3’ (constantly), and has been validated with strong test-retest and inter-rater reliability. The APAI scale has been successfully used to evaluate the effectiveness of interventions for depression symptoms among adolescent survivors of war and displacement in northern Uganda [22-25]. This study uses one depression sub-scale capturing indicators for the local syndrome terms *kumu*. The anxiety subscale captures indicators of the local syndrome term *ma lwor*. The scores on the depression sub-scale range from 0–39 and include questions like, *‘I feel a lot of pain in my heart’,  ‘I sit with my cheek in my palm’*. The scores on the anxiety sub-scale range from 0–36, and include questions like, *‘I think people are chasing me’, ‘I have fast heart rate’.* When the APAI scale was developed and validated by Betancourt et al. in a sample of Acholi adolescents (14–17 years), the internal consistency was strong for both the *kumu* (α = 0.87) and *ma lwor* (α = 0.70) sub-scale. The means from each sub-scale were used to assess differences in mental health status among former abductees who participated in a reintegration process compared to those who have not.

Demographic variables include age, educational attainment, ethnicity, biological children, and orphan status.

### Data analysis

Statistical analyses were performed using SAS (version 9.3). Descriptive statistics (frequencies for dichotomous and categorical variables and medians and interquartile ranges (IQR) for continuous variables) were used to display the distribution of the outcome (mental health), exposure (re-integration status) and other variables. Each abductee reported the year they were abducted, and the abduction frequencies were displayed with a histogram against the historical timeline of the war to explore fluctuations of abduction frequencies against key events in the war including UPDF military operations, the Atiak massacre, the reification of the Amnesty Act, and the Cease-fire signed between the LRA and the Government.

A post-hoc analysis was conducted to test for group differences in mental health status between abductees who accessed a reintegration program versus those who self-reintegrated, a t-test was used to compare the means of the APAI depression sub-scale (*kumu*) and the anxiety subscale (*ma lwor)*. The internal consistency for both sub-scales was assessed using Cronbach alpha coefficients. Less than 5% of cases (n = 6) had missing data on the APAI scale and to avoid case deletion a conservative value of zero was imputed, a technique that biases the results towards the null. A sensitivity analysis was used to assess if there were significant difference between the sample with complete cases (n = 123) compared to the sample with imputed values (n = 129).

The study received ethical approval from the University of British Columbia Behavioural Research Ethics Board, The AIDS Support Organization (TASO) Institutional Review Board (IRB) and the protocol is registered at the Ugandan National Council for Science and Technology (UNCST).

## Results

Among the sample of 400 young women sex workers, a total of 129 (32.2%) women had been abducted by the LRA and were included in this analysis. The characteristics of abductees in this sample are presented in Table 1. Of the 129 abductees, 73 (56.6%) had self-reintegrated and the rest had accessed at least one reintegration program. For the 56 participants who had accessed reintegration programming, the most commonly reported program was a traditional cleansing ceremony (67.9%), followed by receiving amnesty (37.5%), being registered and lodged at a reception centre (28.6%), and receiving a re-insertion package (12.5%). The four reintegration programs are not mutually exclusive, however only 16 of the 56 abductees accessed more than one kind of reintegration program. The cell sizes were not large enough to discern patterns in the combinations of accessed programs.

**Table 1:** **Descriptive statistics of former abductees with and without reintegration programming (n = 129)**^**1**^

**Variables**	**N(%)**^**2**^	**Program reintegration (n = 56)**	**Self- reintegration (n = 73)**
**Demographic variables**			
Age (med. IQR)	22 (20-26)	20.5 (19-25)	23 (20-28)
Tribe: Acholi	124 (96.12)	52 (92.86)	72 (98.36)
Education: <Primary	90 (69.77)	37 (66.07)	53 (72.60)
≥ 1 Biological child	107 (82.95)	41(73.21)	66 (90.41)
Single or Double orphan *(n = 7)*	104 (85.25)	44 (86.27)	60 (84.51)
**Sex work related variables**			
Age at first sex work (med. IQR)	18 (15-20)	17 (16-20)	18 (15-20)
*(n =*^*2*^*)*			
Weekly income from sex work (med.IQR)^3^*(n* =4)	40,000 UGX (20,000-60,000)	40,000 UGX (20,000-60,000)	40,000 UGX (20,000-70,000)
Living with HIV	53(41.09)	31(44.64)	28(38.36)
**War related variables**			
Ever lived in an IDP camp	108 (83.72)	46 (82.14)	62 (84.93)
Age of Abduction (med, IQR)	13 (11-14)	13 (10-14)	12 (11-15)
*(n 8)*			
Year of Abduction (med, IQR)	2002 (1998-2005)	2003 (1999-2006)	2001 (1996-2004)
Period of Abduction			
Less than 3 days	17 (13.18)	5 (8.93)	12 (16.44)
3-14 days	25 (19.38)	7 (12.50)	18 (24.66)
14-60 days	35 (27.13)	18 (32.14)	17 (23.29)
2-l2months	22(17.05)	10(17.86)	12(16.44)
≥ l2months	30(23.26)	16(28.57)	14(19.18)
Child with the LRA	18 (13.95)	11(19.64)	7(9.59)
Manner of departure from LRA			
Escaped	98 (76.56)	45 (80.36)	53 (73.61)
Released	20 (15.63)	7 (12.50)	13 (18.06)
Rescued	9 (7.03)	4 (7.14)	5 (6.94)
Reintegration program^4^			
Traditional cleansing ceremony	-	38 (67.86)	-
Amnesty	-	21(37.50)	-
Reception centre	-	16 (28.57)	-
Re-insertion package	-	7 (12.50)	-

^1^Variables with missing data are noted in parenthesis; ^2^Column Percentages; ^3^40,000 UGX is approximately 15USD; ^4^Categories not mutually exclusive.

The median age of abductees in this study was 22 years (IQR: 20–26) and participants were predominantly from the Acholi tribe (96.1%). Almost 70% had not completed primary education. The majority of the sample (85.3%) were either single or double orphans, with 83.7% having lived in an IDP camp at some point. The median age of abduction was 13 years (IQR:11–14) and when asked how they departed from the LRA, 76.6% of abductees report that they escaped, 15.6% reported that they were released, and 7.0% reported that they were rescued.

**Figure 1:**
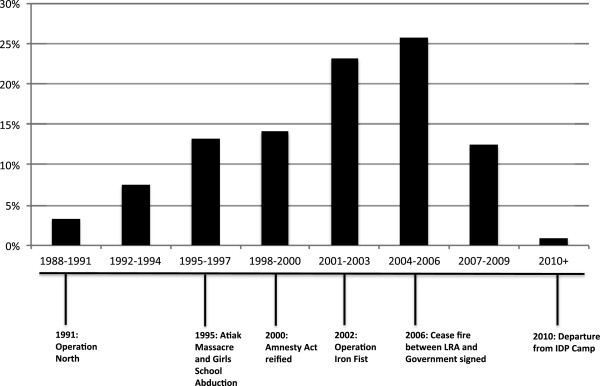
Variation in abduction frequencies over the historical timeline of the war.

Figure 1 displays the frequency of abduction over selected events that punctuate the historical timeline of the war. Participants reported being abducted as early as 1989 and as late as 2010. Almost 25% of the sample reported being abducted in 2004, between Operation Iron Fist (2002) and the 2006 Cease Fire agreement.

The Cronbach alpha revealed that the internal consistency was strong for both the *kumu* (α = 0.76) and *ma lwor* (α = 0.80) sub-scales of the APAI. Overall the total score on the APAI was 20.34 (sd = 8.96). The score for the depression sub-scale *kumu* was 12.84 (sd = 4.79) and anxiety sub-scale *ma lwor* was 8.76 (sd = 5.14). Table 2 displays the mean scores on the APAI total and subscales. Among this sample of abductees, significant differences in mental health status were not detected between those who accessed any kind of reintegration programming compared to those who self-reintegrated. The mean scores on the APAI total, anxiety and depression sub-scales were not significantly different between those who had participated in a reintegration program versus those who self-reintegrated (p < 0.05). Sensitivity analyses reveal that there were no significant difference between participants with missing data and the sample with imputed values.

**Table 2:** APAI descriptive statistics and between group test of means

	**Total**		**Program reintegration**		**Self-reintegration**		**T-test**
	**Mean (sd)**	**Min/Max**	**Mean (sd)**	**Min/Max**	**Mean (sd)**	**Min/Max**	**p-value**
APAI Total	20.34 (8.96)	3/40	20.67 (9.36)	4/40	20.09 (8.69)	3/37	0.562
Depression - *Kumu*	12.84 (4.79)	3/27	13.18 (5.04)	4/27	12.58 (4.60)	3/24	0.484
Anxiety - *Ma Lwor*	8.76 (5.l4)	0/19	8.75 (5.47)	0/19	8.76 (4.91)	0/19	0.990

N.B. Higher scores denote greater burden of mental illness.

## Discussion

This analysis provides descriptive information on the different kinds of reintegration programming that young female abductees accessed in the past and evaluated between group differences in current mental health. The majority of this sample was abducted at a young age (median 13 years), between the years of 1989 and 2010. Almost 25% of participants reported being abducted in 2004, in between Operation Iron Fist in 2002 and the Cease-fire in 2006. This study reveals that just over half (52.7%) of this sample of young abductees were able to access some form of reintegration program, a Figure that is consistent with estimates from other studies [26,27]. The most commonly reported form of reintegration programming was participating in a traditional cleansing ceremony, followed by receiving amnesty, being received at a reception centre, and receiving a re-insertion package. In a post-hoc analysis, significant differences in mental health status were not detected between those who had accessed at least one kind of reintegration program compared to those who self-reintegrated. These results, and the cross-sectional design of this study, are not able to infer that reintegration programming causally affects mental health. However, a contribution that this analysis is able to offer is an exploratory investigation into between group comparisons. In contrast to many studies in the literature that have focused on abductees already registered at receptions centres [27,28], this study utilized the opportunity to compare abductees who accessed reintegration services compared to those who self-reintegrated, but did not find significant differences. Additionally, this study presents information on young female abductees who are less represented in the literature.

Within this sample, a large proportion (56.6%) of abductees self-reintegrated, highlighting the dual challenge of providing wide-reaching reintegration services, and individual-level challenges associated with being able to access the services that were provided and overcome the gendered barriers that make it difficult for women and girls to access DDR programming. One consideration for the gaps in access to reintegration services could be explained by the variation in frequencies of abduction and return over the course of the war. This has been linked to the quantity and quality of services, and influenced the number of ‘reporters’ reaching Reception Centres at different points during the war [27]. For example, as a response to the government led military offensives, Operation North in 1991 and Operation Iron Fist in 2002, the LRA was reported to have increased its frequency of abduction, which is also reflected among this sample of abductees (Figure 1). Important events in the abduction history in northern Uganda were the LRA-led massacre in the village of Atiak where over 300 civilians were murdered for suspected collaboration with the government, and an attack on the Atiak Girls School in 1996 where approximately 60 young women were abducted, a gender specific increase in the frequency of abduction [13]. The current analysis supports this reference as illustrated in Figure 1, where the abductions begin to rise in 1991 and reach their peak just after the cease-fire was signed in 2006. It is reported that peak periods of fighting between the UPDF and the LRA have also been hypothesized to have increased the number of abductees that were able to use the opportunity to escape, thereby creating an influx of returnees at Reception Centres [19,29,30]. Historically, after 2004, the number of returnees decreased and the immediate needs required for disarmament and demobilization began to shift to the long-term needs required for reintegration [19,29,31]. It is very likely that the reintegration experiences among abductees vary depending on the year that they escaped. It is possible that the abductees who escaped before 2002 would have reintegrated during the immediate emergency phase, while those that returned in 2004 and after would have accessed more long-term programming. Within our sample, the average year of abduction among those who accessed reintegration programming was 2003, and those who did not access reintegration programming was 2001. We are not able to examine the association between the number of abductees returning from the LRA and reintegration services among this sample because we only captured the date they were abducted and the length of time they remained in captivity, and not the date they accessed a reintegration program.

Within the sample, over two-thirds of the abductees who accessed a reintegration program, accessed a traditional cleansing ceremony (67.86%). Following a war, affected communities play key roles in cultural and social reconstruction [7], and the high number of abductees able to access a traditional cleansing ceremonies is encouraging. The Survey for War Affected Youth (SWAY) found that only 22% of females had accessed a traditional cleansing ceremony [26], however the SWAY study was conducted in 2005 and it is possible that the current findings reflect the temporal trends in DDR programming over the evolution of the conflict beyond 2005 and perhaps increased effort to include women and girls in DDR programming. An important barrier for traditional ceremonies are the associated costs, particularly for orphans who lack basic resources [19,32]. It is encouraging to see that, despite the large proportion of female single or double orphans (>85%) in this study, many still had access to traditional ceremonies. This is also demonstrative of the efforts that the local community invested in reintegration and reconciliation processes to establish relative peace.

The post-hoc analysis in this study used the APAI scale to measure differences in mental health status between female abductees who accessed a reintegration program compared to those who self-reintegrated. When the APAI scale was developed and validated in the original article by Betancourt et al. [21], the mean score for adolescents (14–17 years) sampled from IDP camps identified with depression through the *kumu* sub-scale was 16.52 (sd = 7.15) and 10.35 (sd = 5.61) for the *ma lwor* sub-scale. Within this sample of sex workers who reported being abducted by the LRA, the mean score for the *kumu* sub-scale was 12.84 (sd = 4.79) and 8.76 (sd = 5.14) for the *ma lwor* sub-scale. This current sample of female abductees has lower scores for both the *kumu* and *ma lwor* sub-scales compared to the sample of youth from IDP camps used during the original validation of the scale. Potential reasons for the lower relative levels of mental illness among this current sample of female abductees could be their older age (mean age 22 years) or the fact that the IDP camps were already disbanded by the time this current study was conducted.

The mental health of abductees is an important topic in conflict-affected populations. A study by McMullen et al., documented higher levels of anxiety and depression among female participants compared to males, and abductees compared to non-abductees [33]. In a study by Pham et al. [5], it was found that 67% of abductees met the criteria for post-traumatic stress disorder (PTSD), and female abductees had higher odds of PTSD (AOR: 8.84, 95% CI:6.07-12.88) and depression (AOR:2.11, 95% CI:1.22-3.63). They also found that going through a reception centre was not significantly associated with PTSD or depression [5], a finding that also mirrors the null results found in this analysis.

One of the goals of DDR programming is to improve psychosocial wellness [3], however this analysis did not find significant differences between these two groups. A good deal of humanitarian aid is invested in reintegration programs with the intention that access to specialized supportive services can improve both short and long-term well-being of abductees and combatants. While this null finding is discouraging, there are several factors that could be contributing to the null finding. For example, high levels of family and community acceptance are linked to lower levels of emotional distress and psychosocial adjustment [34-36]. It is possible that a supportive environment could offset the demand for official reintegration programming. From a temporal standpoint, mental health status was evaluated many years after abduction, and is likely confounded by the long-term challenges associated with reintegration including family reunification, mobilizing and enabling care services, schooling and vocational training [37-40] that extend beyond exposure of access to the four types of reintegration programming examined in this analysis. Additionally, this is a sample of abductees identified through a study on young women involved in sex work. It possible that the current health and social disparities experienced by young sex workers in north Uganda, including high levels of violence, stigma and discrimination and heavy HIV burden, might overshadow the differences that could arise between reintegration programming versus self-reintegration. The findings from this study are insufficient to make claims about the effect of reintegration programs, however they do begin to describe access to programming and offer a preliminary investigation into current mental health status.

The results from this study can lend support to the on-going development of DDR policy and programming in fragile states. The United Nations Department of Peacekeeping Operations has specifically identified the gender gap in evidence to inform programming for women and girls involved in forced conscription. This study found that approximately 45% of female abductees in this sample were able to access a reintegration program including formal processes such as Amnesty International and also Traditional Cleansing Ceremonies. Young women were abducted in their early teens and stayed on average for 12 months in captivity. These findings do support the need to develop programs that are sensitive to both age and gender for women and girls returning from LRA captivity.

### Limitations

This study has several limitations that should be taken into consideration when interpreting these findings. The first relates to sampling issues. Sample selection into the larger study is based on youth and young women engaged in sex work in the last 30 days, and then the analytic sample was then restricted to 129 abductees. Therefore, these findings may not be generalizable to all abductees. However, there is no reason to assume that sex work engagement would differentially impact the two groups of abductees: those who did and did not access reintegration. Additionally, when comparing those who accessed reintegration programs, it is possible that young women can “self-select” into these categories, in that those with intact social support systems and a welcoming family/community may be more able to escape and self-reintegrate, whereas those far from home, with limited family and community support and connections may need to be served by an interim care center in order to find a safe and sustainable placement post-conflict. In this manner, any post hoc comparisons need to better account for these dynamics that may shape self-reintegration vs. supported reintegration.

A second limitation with this analysis is the heterogeneity of the composite reintegration variable. The four different kinds of reintegration programs included in this analysis vary in nature and length. They range from community-based traditional cleansing ceremonies to official amnesty processes. Additionally, each participant self-reported their engagement in each type of reintegration program and this questionnaire did not collect details on their experiences with each program. We did create 3-level variables for each kind of reintegration program (e.g. traditional cleansing ceremony, other reintegration program, no reintegration program) and ran an one-way ANOVAs but found no variance in the APAI score with the alternative categorizations, justifying the decision to collapse all four programs into one variable.

Finally, the association between accessing reintegration programming and mental health is measured cross-sectionally, we do not know the mental health status of abductees prior to their abduction or after their abduction, and can only infer the relationship between reintegration and mental health and not assume causality. It is therefore possible that those women who went through reintegration programming did see some measure of improvement in their mental health as compared to those who self-reintegrated, however we are unable to make this assessment with this sample.

## Conclusions

This analysis of the experiences of young female abductees highlights the barriers to accessing reintegration processes. The young women in this study were abducted into the LRA at a young age, spent an average of one year in captivity, and only half were able to access a reintegration process upon return. This finding highlights the need to mainstream DDR services into the longer-term reconstruction to improve the goals of DDR programming that include opportunities to live ‘normal’ lives, become a functional member of society, resume education, gain skills training, and reduce trauma including anxiety and depression. When young abductees who accessed a reintegration program were compared to those who did not, there was not a significant difference in mental health status. This study begins to elucidate the complex relationships between exiting bush life and reintegration into ‘normal’ society in northern Uganda, and suggests that carefully planned programming, evaluation, and research will benefit our understanding of the legacy of abduction.

## Competing interests

The authors declare that they have no competing interests.

## Authors’ contributions

KAM, GM, MA, MA, KS were involved in data collection and field supervision. KAM and ZP designed the research and wrote the first draft. KAM and PN analyzed the data. TSB, EKB, KS supervised the analysis, interpretation of the data and substantially contributed to the manuscript. KS is the principal investigator and has primary responsibility for the final content. All authors read and approved the final manuscript.
